# Association of Exercise with Inhibitory Control and Prefrontal Brain Activity Under Acute Psychosocial Stress

**DOI:** 10.3390/brainsci10070439

**Published:** 2020-07-10

**Authors:** Manuel Mücke, Sebastian Ludyga, Flora Colledge, Uwe Pühse, Markus Gerber

**Affiliations:** Department of Sport, Exercise and Health, Sport Science Section, University of Basel, 4052 Basel, Switzerland; sebastian.ludyga@unibas.ch (S.L.); flora.colledge@unibas.ch (F.C.); uwe.puehse@unibas.ch (U.P.); markus.gerber@unibas.ch (M.G.)

**Keywords:** Stroop interference, sport, executive function, psychological stress, fNIRS, brain oxygenation

## Abstract

Psychosocial stress has negative effects on cognition in adolescents. The aim of this study was to investigate whether physical exercise can buffer such effects on inhibitory control and associated cortical brain areas. Forty-two male high school students aged 16–20 years and with either low or high exercise levels performed a Stroop task under stress-free conditions and after the Trier Social Stress Test (TSST). Oxygenation of the dorsolateral prefrontal cortex (DLPFC) was measured with functional near-infrared spectroscopy. For inhibitory control, there was no significant primary effect of condition (*F*(1,40) = 1.09, *p* = 303., *ηp²* = 0.027) and no significant condition × group interaction (*F*(1,40) = 2.40, *p* = 0.129, *ηp²* = 0.057). For DLPFC oxygenation, a significant primary effect of condition was observed (*F* (1,38) = 6.10, *p* = 0.018, *ηp²* = 0.138). However, the condition × group interaction (*F* (1,38) = 0.05, *p* = 0.823, *ηp²* = 0.001) remained not significant. Adolescents’ exercise level was not associated with inhibitory control before and after stress. An impact of stress on a neurocognitive level was observed.

## 1. Introduction

Performing cognitively challenging tasks, even under high stress, is of great importance in society [1,2]. For example, success or failure in final exams defines whether a higher level of education can be achieved or not, and work success is often related to dealing with performance pressure and time pressure [3]. In recent years, adolescents have been reported to be at increasing risk for high stress [4]. In a study with 1496 Swiss adolescents, 56% of the participants reported being stressed or overworked often or very often [4]. Since the last few years before graduation are usually perceived as particularly stressful [5], there is a need to examine strategies that have the potential to facilitate the maintenance of cognitive function even under stress.

When a stimulus is appraised as harmful or threatening, a physiological stress response is triggered. Typical real-life situations that involve pressure to perform, uncontrollability, and socio-evaluative threat, such as exams or presentations, usually trigger the highest stress responses (i.e., elevation in cortisol levels) [6]. In laboratory conditions, such situations can be well simulated by the standardized Trier Social Stress Test (TSST), which combines a speech task and a mental arithmetic task performed in front of a critical audience [7]. Under acute stress, physiological resources are mobilized and the organism is prepared for a response [8]. The main stress regulation system involved in this process is the hypothalamic–pituitary–adrenal (HPA) axis. Initiated by the limbic system, the hypothalamus releases corticotrophin-releasing hormone, thereby stimulating the pituitary to release adrenocorticotropic hormone, which causes the adrenal cortex to release cortisol [9]. Cortisol has multiple effects on the human body and brain, and is considered a core stress response parameter [6,9].

Executive functions are higher-order cognitive processes that include working memory, cognitive flexibility, and inhibitory control [10]. These effortful processes are necessary for problem solving, reasoning, and planning, and play a crucial role for adolescents’ mental health and school success [10]. Acute stress largely affects executive functions and associated brain areas [11,12]. However, the magnitude and duration of stress and the specificities of the cognitive task influence the magnitude and direction of stress effects on cognition. While mild stress often facilitates performance in cognitive tasks with low cognitive load, high stress impairs complex cognitive functions that mainly rely on the prefrontal cortex [12]. Recent meta-analytic findings on the effects of acute stress on core executive functions showed that cognitive inhibition (or interference control) in particular is impaired by acute psychosocial stress [13]. Interestingly, Shields et al. reported that this pattern was relatively robust, as it was independent of sociodemographic and study design parameters, and also independent of stress severity, meaning that even under mild stress, interference control can already be impaired [13].

The potential of regular physical activity and exercise to improve executive functioning has been investigated multiple times. Positive effects on brain plasticity and prefrontal gray matter volume have been reported [14,15,16], along with higher behavioral performance in a number of cognitive tasks [17,18]. While effects of multiple exercise modalities were found, coordinative exercise and exercise sessions of longer duration seem to be particularly beneficial [19]. With regard to the effects of exercise on inhibitory control, a brain region of greater interest is the dorsolateral prefrontal cortex (DLPFC). It is known that the DLPFC plays a crucial role in tasks tapping interference control, such as the Stroop task [20,21]. Most commonly, the Stroop task consists of two conditions, where color words are presented in compatible or incompatible ink color, and the response time delay in the incompatible condition corresponds to interference [22]. While other cognitive tasks (e.g., Flanker task) also address interference control, studies have shown that the Stroop task is suitable for the measurement of DLPFC activity with functional near-infrared spectroscopy (fNIRS) [23]. The enhanced performance in more physically active and fitter participants in tasks demanding interference control could be associated with improved sensitivity of the DLPFC to conditions of greater conflict [24], and with a more pronounced left-lateralized activity in the DLPFC [23,25]. While the association of exercise with executive functioning has been studied before, no data are available yet on the association of exercise with executive functioning and activity in relevant brain areas under conditions of increased stress, or directly after stress exposure. Interestingly, as a part of the cognitive control network, the DLPFC has also been shown to be activated during psychosocial stress tasks [26,27,28]. The DLPFC is involved in a negative feedback loop, which (down)-regulates the HPA axis [29]. The other side of the coin is that the DLPFC itself undergoes structural and functional changes when exposed to severe stress [30,31]. Accordingly, regular exercise might have the potential to improve inhibitory control, even under stress, by generally improving the functionality of the DLPFC and thus counteracting detrimental effects of stress on this particular brain region. Another possible mechanism of how exercise could improve executive performance under stress is through a reduction in stress reactivity. Potential positive effects of exercise on stress reactivity have already been demonstrated in an intervention study by Klaperski et al. [32]. A systematic review by Mücke et al. [33] on studies using the TSST showed that around half of the included studies corroborate the notion that exercise is associated with changes in indices of stress reactivity. For this reason, the magnitude of stress reactivity is also taken into account in the present study.

Taken together, in the present study, we aim to investigate the influences of exercise on inhibitory control (more precisely: interference control) and DLPFC oxygenation in the presence of acute psychosocial stress. The DLPFC plays a key role in processing interference control and is influenced by acute stress. Based on the literature, we expected that interference control would deteriorate under acute stress, and that participants who exercised more would show better inhibitory performance under acute stress, in combination with better DLPFC conflict sensitivity and more left-lateralized DLPFC activity (as represented by higher oxygenation), compared to their less active peers.

## 2. Materials and Methods

### 2.1. Participants

Overall, 43 participants were recruited with advertisements, flyers, and personal contact. Only male, generally healthy (non-clinical), right-handed individuals between 16 and 20 years of age were included. The assessment of handedness was based on self-report. We focused on male participants because stress research has shown that mechanisms of stress reactivity are different in men and women, and standardized measurement of stress reactivity in women is challenging because of the influence of the menstrual cycle on cortisol levels [34]. To standardize educational status, which has been shown to influence performance in cognitive tasks [35], only participants currently enrolled in academic high schools were admitted. In order to increase the separation between exercise groups, only adolescents who usually participated in leisure-time exercise and sport activities for (a) less than 1 h per week or (b) more than 6 h per week were eligible for the study. The two cut-offs were chosen because they were considerably below and above the minimum exercise recommendations provided by the American College of Sports Medicine (ACSM) [36]. The duration of leisure-time exercise and/or sport activities per week was assessed via self-report, with exercise being defined as regular activities in the past four weeks that caused sweating and getting out of breath, and lasted longer than 30 min. Self-reported exercise levels were used to allocate participants to the low exercise group (<1h/week) or high exercise group (> 6h/week) and were verified with accelerometry (see Section 2.4). At least 3 days before data assessment, participants were informed about the study procedures and provided written informed consent. All study procedures were performed in accordance with ethical principles of the Declaration of Helsinki and were approved by the local ethics committee (Ethikkommission Nordwest- und Zentralschweiz; EKNZ number 2017-01330, Basel, Switzerland) before the start of the study.

### 2.2. Instruments

For the induction of psychosocial stress, the TSST was used, and saliva cortisol samples were collected to measure the reaction of the HPA axis to the stressor. Both procedures are described in Section 2.6. Inhibitory control was measured with a computerized version of the Stroop task, with simultaneous measurement of DLPFC activity with fNIRS. Details on the cognitive task and DLPFC measurement are presented in Section 2.5. With regard to control variables, body height and weight were measured via a stadiometer and an electronic scale (Tanita BC-601, Tokyo, Japan), respectively, and participants filled in a questionnaire that included socioeconomic status (1 item) and psychological variables. As psychological control variables, chronic stress (perceived stress scale (PSS)) [37], sleep complaints (insomnia severity index (ISI)) [38], and psychopathology (Strengths and Difficulties Questionnaire (SDQ)) [39] were assessed, since these parameters could potentially influence executive functioning. All psychological instruments showed acceptable internal consistency in the present sample (Cronbach’s alpha values for the PSS = 0.75, for the ISI = 0.65, and for the SDQ = 0.72), although it should be noted that in the ISI, Cronbach’s alpha was slightly below the often-used threshold of 0.7 [40]. Participants’ physical activity was measured with accelerometers over the course of 7 days (see Section 2.4).

### 2.3. Procedure

**Figure 1:**
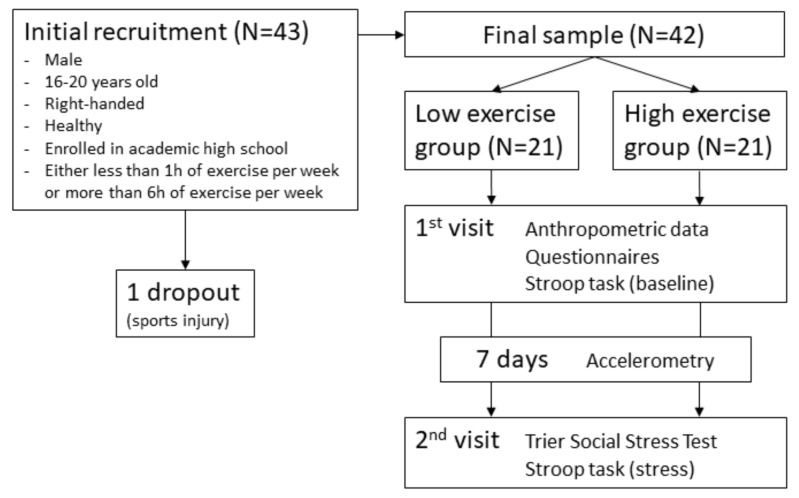
Study procedure.

Figure 1 gives an overview of the study procedures. To minimize the potential influence of variations in diurnal cortisol levels [41], all study appointments were scheduled in the afternoon and started either at 13:00 or at 16:00. At the first appointment, anthropometric data were collected and participants filled in questionnaires. Subsequently, the participants performed a computerized Stroop task under stress-free conditions (C1) while wearing an fNIRS head cap (see Section 2.5).

One week later, at the same time of the day, participants were scheduled for the second study appointment, consisting of the Trier Social Stress Test (TSST) and the Stroop task (C2). Prior to the appointment, participants were instructed to not engage in physical exercise and to refrain from drinking alcohol or coffee and taking any medication during the 24 h before the appointment, to refrain from eating and drinking (except water) during the hour before the appointment, and to avoid rushing to the appointment [42]. Upon arrival, participants rested for 10min in order to reduce the influences of possible stress factors before and/or during arrival. Subsequently, the fNIRS head cap was applied. Then, the TSST was performed as described in Section 2.6. To determine stress reactivity, saliva cortisol was measured after the TSST preparation phase and directly after the Stroop task. Additionally, participants completed a brief questionnaire on psychological stress parameters before and after the TSST. Directly after the TSST, the Stroop task was performed as in C1. This design allows for the comparison of inhibitory performance and DLPFC oxygenation under stress (C2) with both parameters under non-stressful conditions (C1).

Between both study appointments, participants wore accelerometers on the hip for 7 consecutive days (see Section 2.4). After debriefing, all participants received financial compensation for their participation in the study.

### 2.4. Accelerometry

Physical exercise and physical activity are related constructs [43]. To validate the self-reported exercise levels, and to make sure that participants who reported low exercise levels also showed lower physical activity levels (and vice versa), each participant’s physical activity was monitored objectively via waist-worn, triaxial accelerometer (ActiGraph wGT3X-BT, Actigraphcorp, Pensacola, USA) over the course of 7 consecutive days. Non-wear time was determined using the Troiano algorithm [44]. Measured days were considered valid if the device was worn for at least 600 min per day. Total measurements were considered valid if ≥1 valid weekend day, ≥3 valid week days, and ≥5 valid days in total were found [45], leading to the exclusion of the accelerometry data of two participants (one in each group). For calculation of physical activity, an algorithm introduced by Freedson et al. was used, distinguishing between sedentary, light, moderate, vigorous, and very vigorous physical activity [46]. Finally, moderate-to-vigorous physical activity (MVPA) and vigorous physical activity (VPA) were calculated.

### 2.5. Cognitive Task and Prefrontal Brain Activity

Procedures presented in this section are described in detail in Ludyga et al. [22] and are only summarized here. Assessments took place in a dimly lit room at a temperature of 21–22 °C. Ambient noise was reduced to a minimum and participants were instructed to avoid head movements and speaking during the task. A computer-based version of the Stroop Color and Word task was conducted to assess the inhibitory component (more precisely: interference control) of executive functioning [47]. Computer-based versions have been found to be of high test–retest reliability and they also produce a Stroop-effect comparable to the original pen and paper version [48]. Additionally, a review supports the validity of this task as a measure of executive function and this result was consistent across different variants of computerized Stroop tasks [49]. The employed version consisted of compatible and incompatible trials. In compatible trials, color words were presented in the same ink color (e.g., “blue” printed in blue), whereas in incompatible trials, color words appeared in a different color of ink (e.g., “green” printed in yellow). To ensure similar visual content, the German color words “blau” (blue), “gelb” (yellow), and “grün” (green) were used. Participants were instructed to press a button corresponding to the color of ink, ignoring the meaning of the word they read, and to react as quickly and accurately as possible. The task included six test blocks, which contained 36 trials each. Resting periods between the blocks lasted 30 s. Before testing, two practice blocks with 24 trials each were conducted. Compatible and incompatible test blocks alternated. Within each block, the stimuli were presented with equal probability and in a fully randomized order. For analysis, an interference score was calculated as the difference between reaction time (of response-correct trials) on incongruent trials minus reaction time on congruent trials. A lower interference score equals higher inhibitory control. Additionally, mean reaction time and accuracy were extracted separately for both compatible and incompatible trials to examine whether possible group differences were influenced by a speed-accuracy trade-off.

For measurement of prefrontal brain oxygenation, a dual-wavelength (760 and 850 nm) continuous-wave fNIRS system with a sampling rate of 7.8125 Hz (NIRSport, NIRx Medical Technologies, Berlin, Germany) and the recording software NIRStar 15.0 (NIRx Medical Technologies, Berlin, Germany) was used. Sixteen optodes (8 illumination sources, 8 light detectors) were attached to a flexible cap, which was then fitted to the participant’s head. Optodes were distributed over the prefrontal cortex, resulting in a total of 23 channels (montage design; see [22]). Spacers were used to keep the inter-optode distance constant at 3 cm, which is considered to be the best trade-off between high light penetration depth and sufficient signal-to-noise ratio [50,51]. To prevent ambient light from affecting the measurements, participants additionally wore a loosely-attached, optically-opaque overcap. During preparation, signal quality was assessed. Recording procedures were in line with previously established quality standards [52] and recommendations for fNIRS assessments in exercise-cognition research [53].

After recording, fNIRS data were processed with Homer2 version 2.3 [54]. The processing stream followed the one proposed by Brigadoi et al. [55] and is described in detail in Ludyga et al. [22]. Artifacts exceeding defined thresholds were automatically marked and manually verified. Based on the results of systematic comparisons of artefact correction techniques [56,57], spline interpolation was applied to correct marked artefacts, followed by a frequency filter with a low cut-off at 0.01 Hz and a high cut-off at 0.5 Hz [58,59,60]. Block averages were created for compatible and incompatible trials with the 5 s period preceding the test block used as reference. For this publication, only channels representing left and right DLPFC were used. For each side, the average of 4 channels was calculated because previous studies found test-retest reliability to be higher at cluster level compared to individual optodes [61].

### 2.6. Stress Induction and Measurement of Stress Reactivity

Psychosocial stress was induced using the TSST [7]. The TSST is composed of a mock job interview and a mental arithmetic task, which are both performed in front of a committee. This combination of a motivated performance task with the additional element of uncontrollability and socio-evaluative threat has been shown to be more effective in triggering a physiological stress response than other laboratory stressor tasks [6]. Participants were instructed to envision a situation in the near future in which they had finished school and were being offered a job interview for their dream job. The committee for the job interview consisted of two persons (one male and one female) and was introduced to the participants as the manager of the company and an associate who is specifically trained in the observation and interpretation of body language and voice frequency. After the introduction and a 10 min preparation phase, participants performed a 5 min free speech facing the committee, which was followed by a 5 min mental arithmetic task. Throughout the speech, the committee showed neutral facial expressions and only used standardized responses if required (e.g., “You still have time left. Please continue.”). The mental arithmetic task contained five rounds of counting backwards as quickly as possible in steps of 9, 11, 7, 13 and 8, respectively. In case of miscalculation, the participant was interrupted and asked to begin again at the last correctly calculated number.

Cortisol collected from saliva samples represented the stress reactivity of the HPA axis, the main physiological stress regulation system. Salivary cortisol levels rise with about 10 min delay relative to stressor onset [62]. Accordingly, saliva samples (Salivette^®^ Blue cap, Sarstedt, Nümbrecht, Germany) were collected after the 10 min preparation phase (S1) and directly after the Stroop task (S2). Cortisol reactivity was defined as the value of S2 minus S1. Samples were stored at −20 °C and sent to the Biochemical Laboratory of the University of Trier, Germany, where time-resolved fluorescence immunoassay was applied to analyze cortisol concentrations (in nmol/L).

### 2.7. Statistical Analysis

For sample size calculation, an a priori power analysis was calculated with G*Power. As the association between exercise and inhibitory control under stress has not been investigated before, a calculation based upon the existing literature was not possible. Therefore, we decided to assume a medium effect size of f = 0.25 and to use the knowledge gained through our study for sample size calculations in future studies. With the parameters “repeated measures analysis of variance (ANOVA),” α-error probability = 0.05, power = 0.80, number of measurements = 2, and correlation among repeated measures = 0.5 for inhibitory control, power calculation resulted in a required minimum sample size of *n* = 34.

As a manipulation check, effectiveness of the TSST was measured using a repeated measures ANOVA on cortisol response. Because no group differences with regard to cortisol reactivity to the stressor were found, subsequent analyses were performed without controlling for cortisol.

For both inhibitory performance and DLPFC oxygenation, group differences in baseline values (C1) were examined using independent *t*-tests. To determine the influence of exercise on inhibitory performance under stress, a repeated measures ANOVA was calculated with Stroop interference in reaction time at C1 and C2 as within-subject factors and exercise group as a between-subject factor.

DLPFC activity related to inhibitory control was defined as the mean oxygenated hemoglobin in response to incompatible test blocks minus mean oxygenated hemoglobin in response to compatible test blocks of the Stroop task (Δ_OXY_). Since studies found indications for different activation patterns in left and right DLPFC [21,63], both hemispheres were included in analysis separately. Thus, the influence of exercise on DLPFC oxygenation under stress was analyzed using a repeated measures ANOVA with condition (Δ_OXY_ at C1 and C2) and hemisphere (Δ_OXY_ of left versus right hemisphere) as within-subject factors and exercise group as a between-subject factor.

For all repeated measures ANOVAs, Greenhouse–Geisser-corrected main effects and interactions were reported. Effect sizes were classified as small (*d* ≥ 0.2; *ηp²* ≥ 0.01), medium (*d* ≥ 0.5; *ηp²* ≥ 0.06), or large (*d* ≥ 0.8; *ηp²* ≥ 0.14) [64]. Significance level was defined as *p* < 0.05 and all statistical computations were performed with SPSS 24 (IBM Corporation, Armonk, NY, USA).

## 3. Results

### 3.1. Sample Description

After C1, one participant dropped out because of a sports injury. Therefore, only data of the remaining 42 participants were analyzed. Anthropometric, psychometric, and accelerometry data are presented in Table 1. Additional information on correlations of the control variables with the main outcomes is provided in the Supplementary Materials (Table S1). For verification of the group separation based on self-report, independent samples *t*-tests were calculated and showed that the groups significantly differed in VPA and MVPA (Table 1). None of the control variables showed significant group differences at baseline (Table 1).

### 3.2. Effectiveness of the Stressor

The average cortisol level at baseline was 3.5 (standard deviation 1.7) nmol/L. After the TSST, cortisol levels rose to 9.3 (5.1) nmol/L. In the low exercise group, cortisol levels were 3.8 (1.9) nmol/L at baseline and 10.1 (5.7) nmol/L after the stressor, compared to 3.1 (1.4) nom/L and 8.4 (4.5) nmol/L in the high exercise group. Repeated measures ANOVA showed a significant and strong effect of condition *F*(1,40) = 60.99, *p* < 0.001, *ηp²* = 0.604), but no significant condition × group interaction (*F*(40) = 0.46, *p* = 0.500, *ηp²* = 0.011).

### 3.3. Inhibitory Performance

**Figure 2:**
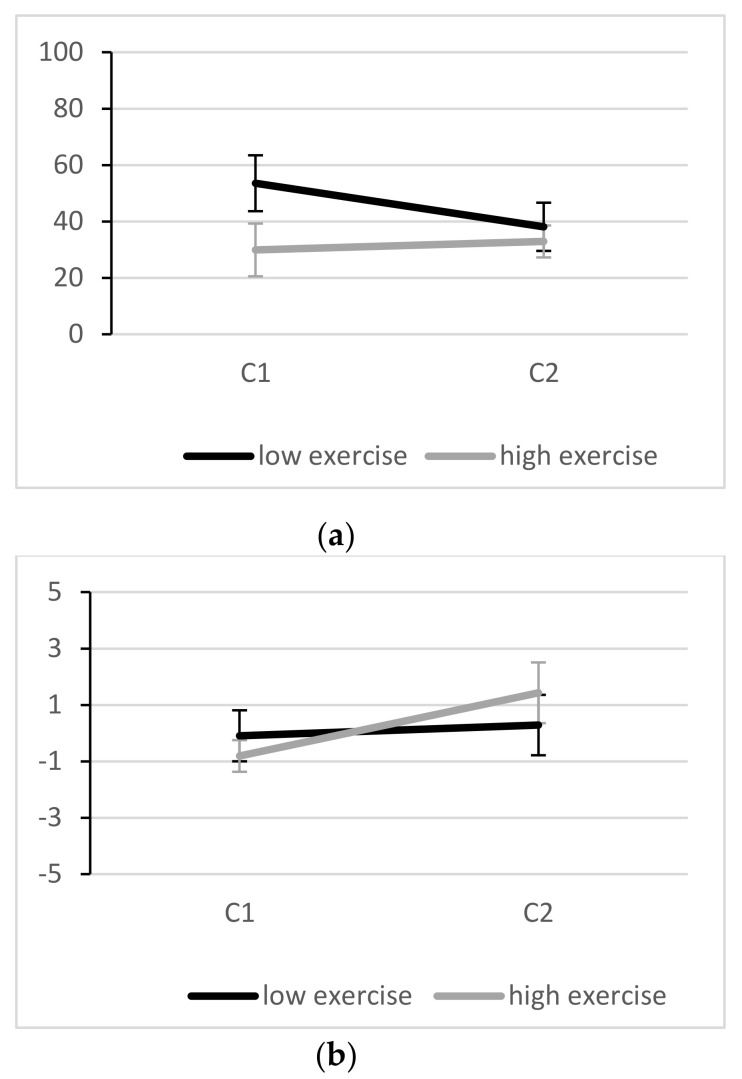
Stroop interference scores (incompatible minus compatible trials) for reaction time ((**a**) in ms) and accuracy ((**b**) in %) in the low and high exercise group. Error bars are standard errors of the mean.

Changes in Stroop interference are depicted in Figure 2a. At C1, average reaction time interference in the low exercise group was 53.5 (45.4) ms, compared to 29.9 (39.2) ms in the high exercise group. An independent *t*-Test revealed no baseline (C1) differences between groups (*t* (40) = 1.81, *p* = 0.078, *d* = 0.57). However, it should be noted that a medium effect size indicated lower (better) interference scores in the high exercise group compared to the low exercise group. At C2, interference scores of 38.1 (42.9) ms and 32.9 (25.9) ms were observed, respectively. To investigate whether potential group differences are related to speed-accuracy trade-offs, we also analyzed response accuracy (Figure 2b). No group differences were present with regard to response accuracy at C1 or C2 for compatible (C1: *p* = 0.999, C2: *p* = 0.951) or for incompatible trials (C1: *p* = 0.739, C2: *p* = 0.498).

The repeated measures ANOVA revealed no significant primary effect of condition (*F*(1,40) = 1.09, *p* = 303, *ηp²* = 0.027) and no significant interaction between condition and exercise group (*F*(1,40) = 2.40, *p* = 0.129, *ηp²* = 0.057). However, for the latter a small-to-medium effect size can be observed (see 4.1).

### 3.4. Oxygenation of Left and Right DLPFC

**Figure 3:**
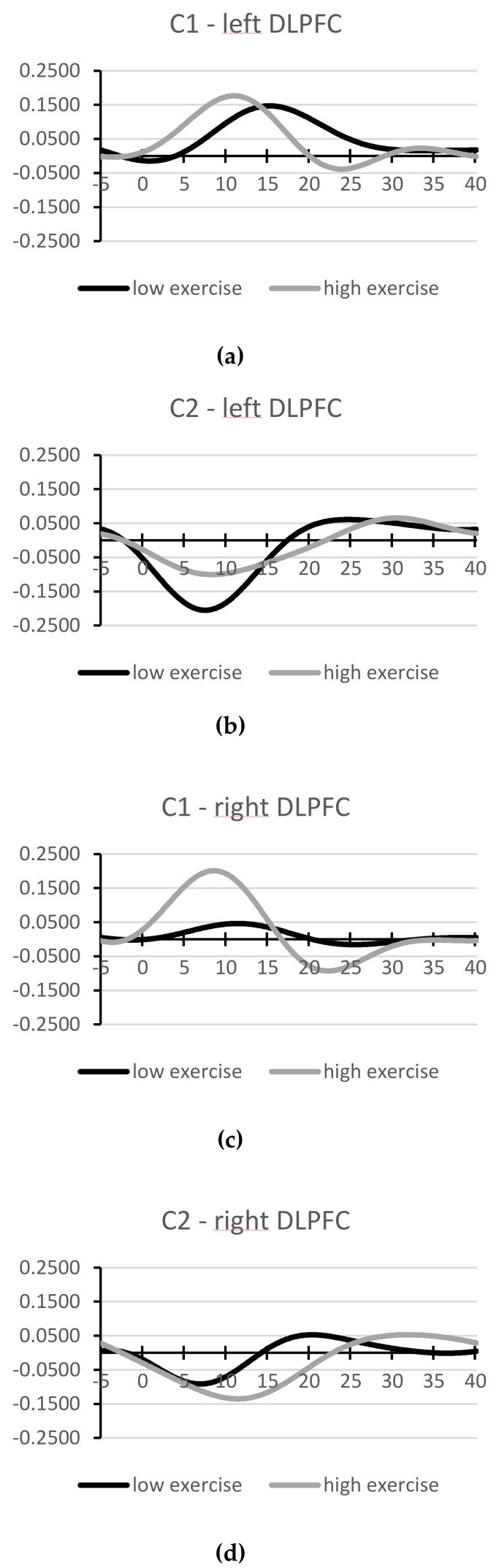
FNIRS interference waveforms (averaged oxygenation during incompatible test blocks minus compatible test blocks; in mmol/L) of the left dorsolateral prefrontal cortex before (a) and after the stressor (b), and of the right dorsolateral prefrontal cortex before (**c**) and after the stressor (**d**).

After signal processing, two participants were excluded from further fNIRS analysis because of overly noisy data. Of the remaining 40 participants, average fNIRS waveforms corresponding to both exercise groups’ interference waves (incompatible minus compatible Stroop condition) are depicted in Figure 3. A more detailed image of the averaged waveforms during incompatible and compatible test blocks can be found in the Supplementary Materials (Figure S1).

An independent *t*-test indicated no baseline (C1) differences between groups for Δ_OXY_ of left (*t*(38) = −0.67, *p* = 0.509, *d* = 0.22) and right hemisphere (*t*(38) = −1.40, *p* = 0.174, *d* = 0.46). Recent fNIRS research suggested a leading role of the left DLPFC in tasks demanding inhibitory control [23]. In our sample, when comparing oxygenation interference in both hemispheres at C1, this was only true for participants with lower exercise (*t*(20) = 2.09, *p* = 0.049, *d* = 0.46), but not for those with higher exercise (*t*(18) = 0.37, *p* = 0.741, *d* = 0.08).

The repeated measures ANOVA showed a significant and strong main effect of condition (*F*(1,38) = 6.10, *p* = 0.018, *ηp²* = 0.138), indicating a shift towards lower relative oxygenation during incompatible test blocks and higher relative oxygenation during compatible test blocks after stress induction in both exercise groups (see Figure 3 and Supplementary Materials, Figure S1). No main effect of hemisphere was observed (*F* (1,38) = 1.11, *p* = 0.299, *ηp²* = 0.028). All interaction terms were not statistically significant (*p* > 0.307). No correlation between DLPFC lateralization and Stroop interference was found at C1 or C2 (*p* > 0.514).

## 4. Discussion

The aim of this study was to investigate whether regular exercise is associated with inhibition (interference control) and corresponding activity in the DLPFC under acute stress. The main findings were that (a) no systematic differences between high and low exercise group were observed with regard to behavioral inhibitory control and DLPFC oxygenation patterns under stress; (b) both groups showed comparable cortisol reactivity to the psychosocial stressor; (c) compared to the stress-free condition, interference control did not change under stress; and (d) across all participants, DLPFC activity was altered under stress, with higher relative oxygenation during compatible test blocks and lower relative oxygenation during incompatible test blocks compared to the pre-stress condition. On a side note, potential group differences in the pre-stress condition occurred: for behavioral inhibitory control, medium effect sizes indicated higher performances in participants with higher levels of exercise.

### 4.1. Associations with Exercise

Our results show no statistically significant association between regular exercise and inhibitory performance under enhanced psychosocial stress. In previous studies investigating this association under stress-free conditions, exercise and fitness have consistently been shown to be positively associated with PFC functioning and cognitive performance [18,65]. Furthermore, research has suggested that stress generally has negative effects on the prefrontal cortex and executive functioning [11], although this relationship might be more complex [13]. We therefore hypothesized that exercise might be able to buffer negative effects of stress on executive functioning. However, our data do not indicate such differences between frequently exercising and inactive adolescents. Possible reasons for non-significant findings could be an insufficient stressor or too little ego-involvement in the stress task. Some of the study participants had never attended a job interview before the study, so the TSST does not represent their current life situation. However, the TSST elicited highly significant increases in saliva cortisol in the participants, indicating the activation of the HPA axis in response to the stressor, and they reported significant increases in psychological stress parameters as well (data not shown). Therefore, it is unlikely that the lack of change in inhibitory control from the stress-free condition to the TSST was due to insufficient stress induction. Another possible issue is the group separation with respect to exercise. Our recruitment strategy of only including participants with self-reported exercise of <1 h or >6 h per week ensured sufficient separation and was verified by significant group differences in MVPA and VPA. However, as the low exercise group showed relatively high MPVA levels (see Table 1), it is possible that the group differences were too little to produce an effect. Furthermore, although exercise has previously been related to executive function, some findings suggest that (fitness and motor) skills targeted by exercise may explain this relation [66]. Other researchers reported that besides fitness, game skills in team sports and aspects of fine motor control predicted inhibitory control, showing that exercise seems to benefit inhibitory control through several pathways [67]. Thus, comparing changes in inhibitory control across conditions between groups differing in motor skills, fitness, and other exercise-related skills might have yielded different results. Additionally, it cannot be ruled out that the stress-buffering role of exercise might only be observable in highly chronically stressed people [68]. Participants in our sample reported relatively low chronic stress levels, and none of them could be classified as highly chronically stressed. It is possible that it is not a single stressor, but repeated and high chronic stress that leads to substantial functional changes in the PFC, and that this condition is required for the buffering effects of exercise on inhibitory performance to be observed [31].

If we extend the discussion of our data to results based on effect sizes, a small-to-medium effect in the condition by exercise group interaction (*ηp²* = 0.057) indicated potential group differences across conditions, with a tendency towards an improvement in inhibitory control from C1 to C2 in participants with lower exercise, while participants with higher exercise showed approximately constant scores from C1 to C2 (see Figure 2a). Considering that a medium effect size indicated that more active participants performed better than their inactive peers at baseline (*d* = 0.57), which is well in line with the current literature [18], one could speculate that while adolescents with low exercise levels show relatively high Stroop interference under non-stressful conditions and manage to improve under stress, adolescents with higher exercise levels perform at a higher level under both conditions and are less affected by acute stress. Byun et al. [58] showed that under certain circumstances, Stroop performance and arousal level can be positively related, which might have been the case in more inactive participants in our sample. However, more research is needed to support or discount this preliminary finding.

With regard to DLPFC activity, we hypothesized that we would find better DLPFC conflict sensitivity (that is: lower oxygenation during compatible and higher oxygenation during incompatible Stroop blocks) and more left-lateralized DLPFC activity in the high exercise group, compared to their less active peers. Consistent with the results on behavioral performance, we did not find any systematic differences in DLPFC conflict sensitivity between high and low exercise groups. Furthermore, no systematic group or condition effects with regard to DLPFC hemisphere were observed. Other researchers reported associations of better Stroop performance with left-lateralized DLPFC activity [23], especially in participants with higher physical fitness [25]. DLPFC lateralization and Stroop performance were not associated in our sample. Perhaps exercise and fitness-related differences in DLPFC lateralization during interference control tasks are age-dependent. The HAROLD phenomenon describes the reorganization of the brain due to age-related structural and physiological decline, resulting in less lateralized brain activity during cognitive tasks [25]. While exercise might have the potential to counteract these changes by delaying the age-related decline, no such effects can be observed in young people. Moreover, Vanderhasselt et al. [21] argued that lateralization effects during the Stroop task are largely influenced by the specificities of the protocol used.

Finally, a significant primary effect of condition indicated that DLPFC oxygenation was altered under stress. That is, across the whole sample, activity during compatible blocks increased and activity during incompatible blocks decreased under stress (in comparison to the stress-free condition). This pattern was more pronounced in the left DLPFC. According to Vanderhasselt et al. [21], during response conflict, the anterior cingulate cortex is activated, leading to recruitment in the DLPFC for increased cognitive control in the task. In our data, this mechanism is represented by the positive interference waves (Figure 3) at baseline, indicating higher DLPFC activity when interference control was necessary. However, under stress, different patterns emerged: during compatible test blocks, which require attention control but no inhibitory performance, DLPFC oxygenation increased, whereas during incompatible test blocks, which require the inhibition of a prepotent response (suppression of word reading), DLPFC oxygenation decreased. This activation pattern suggests a decreased capacity for higher order cognitive function under stress. According to Arnsten [11], p. 415, under acute stress the amygdala initiates high levels of catecholamine release, which, in synergy with increases in glucocorticoid levels, “switch the brain from thoughtful, reflective regulation by the PFC to more rapid reflexive regulation by the amygdala and other subcortical structures.” However, in our sample, this change in activation patterns had no effect on behavioral performance. One possibility for this unexpected result is that Stroop performance at C2 is confounded by learning effects. However, this is unlikely since C1 and C2 were seven days apart, and in both appointments, two exercise rounds for the compatible and incompatible conditions were performed. Two other mechanisms seem more likely. On the one hand, it might be possible that due to insufficient task difficulty or duration, no effect of changes in DLPFC oxygenation on inhibitory performance can be observed yet. Plieger et al. [69] showed that stress-related changes in cognitive performance depend on cognitive load. With higher cognitive load, the observed changes in DLPFC oxygenation under acute stress might result in reduced inhibitory performance. On the other hand, the observed changes in DLPFC activation might not originate directly from task-related inhibitory processes but from other stress-relevant processes that are monitored in the DLPFC, such as emotion regulation. Ochsner et al. [70] showed that positive reappraisal of negative situations is accompanied by enhanced activity in the left DLPFC. Other studies reported similar involvement of the DLPFC in emotion regulation [71,72,73].

### 4.2. Inhibitory Control Under Stress

The results of our study further suggest that in later stages of adolescence (16–20 years of age in our sample), interference control is neither impaired nor enhanced by acute psychosocial stress. Similarly, Ishizuka, Hillier, and Beversdorf [74], who administered a verbal version of the Stroop task during cold water hand submersion in undergraduate students, found no significant difference to the control condition. In their study on the effect of stress on selective attention, Chajut and Algom [75] administered several versions of the Stroop task. Psychosocial stress was manipulated with psychometric tasks, which had to be performed with or without increased task difficulty, time pressure, and threat to the ego. Interestingly, in their sample of 160 university freshmen aged 20 to 25 years, they observed that under low stress, task performance was affected by task-irrelevant variations, while under high stress, focus on the target attributes was improved. Accordingly, stress was associated with a reduced Stroop interference and an increased inhibitory performance in their study. Studies using other tasks measuring inhibitory control also came to contrasting results. Schwabe, Hoffken, Tegenthoff, and Wolf [76] tested 72 university students and showed improved performance in a stop-signal task after a socially evaluated cold pressor task (SECPT). However, in a similar sample of 97 undergraduate students, Roos et al. [77] found impaired performance in a stop-signal task after the TSST. Similar to the latter, Sänger et al. [78] and Vinski and Watter [79] found impaired performance in cognitive inhibition after the SECPT and the TSST, respectively.

Several factors are discussed as potential causes for these differences in the effect of stress on inhibitory control tasks. In their meta-analysis on the effect of stress on core executive functions, Shields et al. [13] included participants’ sex and age, type and severity of stressor, time delay from stressor to inhibition task, outcome type (reaction time versus accuracy based), and inhibition type (response versus cognitive inhibition) as potential moderators. Interestingly, their results show differential effects only with regard to inhibition type: acute stress impairs cognitive inhibition (β = −0.21, *p* = 0.021)—which Shields et al. [13] used interchangeably with interference control—and enhances response inhibition (β = 0.30, *p* = 0.041). No influence from other moderators was found. Given that according to Nigg [20] the Stroop task is a classic interference control task, negative effects of acute stress on task performance can be expected. However, this was not the case in our study. One reason might be the particularities of the adolescent sample. However, Shields et al. [13] reported no moderating influence of age. In earlier work, an inverted U-shaped relationship between severity of stress or arousal and cognitive performance has been proposed as an explanation [80]. But again, this notion is not supported by more recent meta-analytical findings [13]. Another possibility is that the influence of stress depends on task difficulty, meaning that while in relatively simple tasks, effects might be absent, performance in more complex tasks might be impaired by stress. In conclusion, more research is needed on the factors underlying the heterogeneous effects of acute stress on inhibition.

### 4.3. Strengths and Limitations

The primary strengths of our study are (i) the use of an effective and validated stress task which is currently considered as the gold-standard for stimulating neuroendocrine stress responses [62], (ii) a thorough fNIRS data analysis procedure following recent methodological recommendations [53,55], (iii) the verification of the group allocation (high vs. low exercise group) via accelerometry, and (iv) the consideration of major covariates, such as age, BMI, socioeconomic status, and sleep complaints.

The results of this study should be interpreted in light of several limitations. Because our study focused on male adolescents aged 16–20 years, further research on female adolescents and other age groups is necessary. Other inclusion criteria (e.g., regarding educational or socioeconomic status) could have yielded different results. Since no control condition was used in our study, learning effects in the Stroop task from C1 to C2 cannot be fully precluded. However, this is very unlikely, since both appointments were one week apart and during both appointments, exercise trials were performed before testing for both conditions. While our study is a first attempt to examine the association of exercise with inhibitory control and prefrontal brain activity under acute psychosocial stress, our data did not provide support for such an association (i.e., no condition X group interaction). Nevertheless, we acknowledge that our study design does not allow conclusions on whether the lack of any association between outcomes can be generalized to other stressors and other inhibitory control tasks, and the results do not necessarily extend to other executive functions. With regard to the measurement of physical activity via accelerometry, we acknowledge that some types of exercise (e.g., activities in the water or static exercise) are difficult to assess with accelerometry. Additionally, it remains unclear whether a comparison of specific exercise types rather than the total dose would have produced different results. Lastly, our study design does not allow causal inferences with regard to the effect of physical exercise on inhibitory control under stress. Therefore, intervention studies are needed to find out whether executive functioning can be improved with regular exercise training.

### 4.4. Conclusions

Our study suggests that in healthy male adolescents with higher educational status, acute psychosocial stress does not seem to affect behavioral inhibition. Furthermore, regular exercise was not associated with changes in inhibitory control or DLPFC activity under acute stress. Consequently, our results suggest that the maintenance of high exercise levels does not promise improved inhibitory control under exposure to psychosocial stress in male adolescents. Further studies should consider whether a stress-buffering effect is present in adolescents suffering from chronic stress.

## Figures and Tables

**Table 1:** Sample characteristics of high and low exercise groups.

	Low Exercise Group (*n* = 21)		High Exercise Group (*n* = 21)		
	Mean	SD	Mean	SD	T
Age (years)	17.2	1.1	17.1	1.2	0.14
BMI (kg/m^2^)	22.9	5.1	22.0	2.1	0.81
Socioeconomic status	3.1	0.6	3.5	0.8	−2.01
Sleep complaints (ISI)	6.3	3.7	5.9	4.1	0.28
Chronic stress (PSS)	14.3	5.4	13.3	3.6	0.67
Psychopathology (SDQ)	14.8	9.5	13.5	4.5	0.58
VPA (min/day)	6.4	8.6	15.0	7.3	−3.38 *
MVPA (min/day)	60.6	22.4	83.3	20.0	−3.39 *

ISI = insomnia severity index, MVPA = moderate-to-vigorous physical activity, PSS = perceived stress scale, SDQ = Strengths and Difficulties Questionnaire, VPA = vigorous physical activity, * *p* < 0.05.

## Supplementary Materials

The following are available online at https://www.mdpi.com/2076-3425/10/7/439/s1, Table S1: Zero-order Pearson correlations of the control variables with the main outcomes, Figure S1: FNIRS waveforms of left and right dorsolateral prefrontal cortex during compatible (com) and incompatible (inc) test blocks without stress (C1) and after the stressor (C2) in participants with high and low exercise levels (in mmol/l).,
