# Supplementary material for: Association of Exercise with Inhibitory Control and Prefrontal Brain Activity Under Acute Psychosocial Stress
Source: Brain Sci. 2020 Jul 10;10(7):439. doi: 10.3390/brainsci10070439 (PMC7408469; doi:10.3390/brainsci10070439)
Supplement: Supplementary file 1 [file brainsci-10-00439-s001.pdf]

Supplementary Material.

Table S1. Zero-order Pearson correlations of the control variables with the main outcomes.

		1	2	3	4	5	6	7	8	9
1	Age (years)									
2	BMI (kg/m^2)	.27								
3	SES	.06	.03							
4	ISI	.15	.24	-.04						
5	PSS	.21	.13	.06	.07					
6	SDQ	.22	.32*	.04	.23	.58**				
7	MVPA	.13	.37*	.09	-.12	-.02	.03			
8	VPA	-.11	.06	.25	-.29	.04	.02	.64**		
9	Cortisol reactivity (S2-S1)	.02	.05	-.18	.28	-.13	-.02	.00	-.06	
10	Stroop interference (C2-C1)	-.15	.01	.09	-.17	.08	.15	.00	.09	-.06
11	fNIRS interference (C2-C1)	-.02	-.19	-.04	.00	-.04	-.08	-.03	-.03	-.08

ISI=Insomnia Severity Index, MVPA=Moderate-to-vigorous physical activity, PSS=Perceived Stress Scale, SDQ=Strengths and Difficulties Questionnaire, SES=Socioeconomic status, VPA=Vigorous physical activity; * $p < 0.05$, ** $p < 0.01$

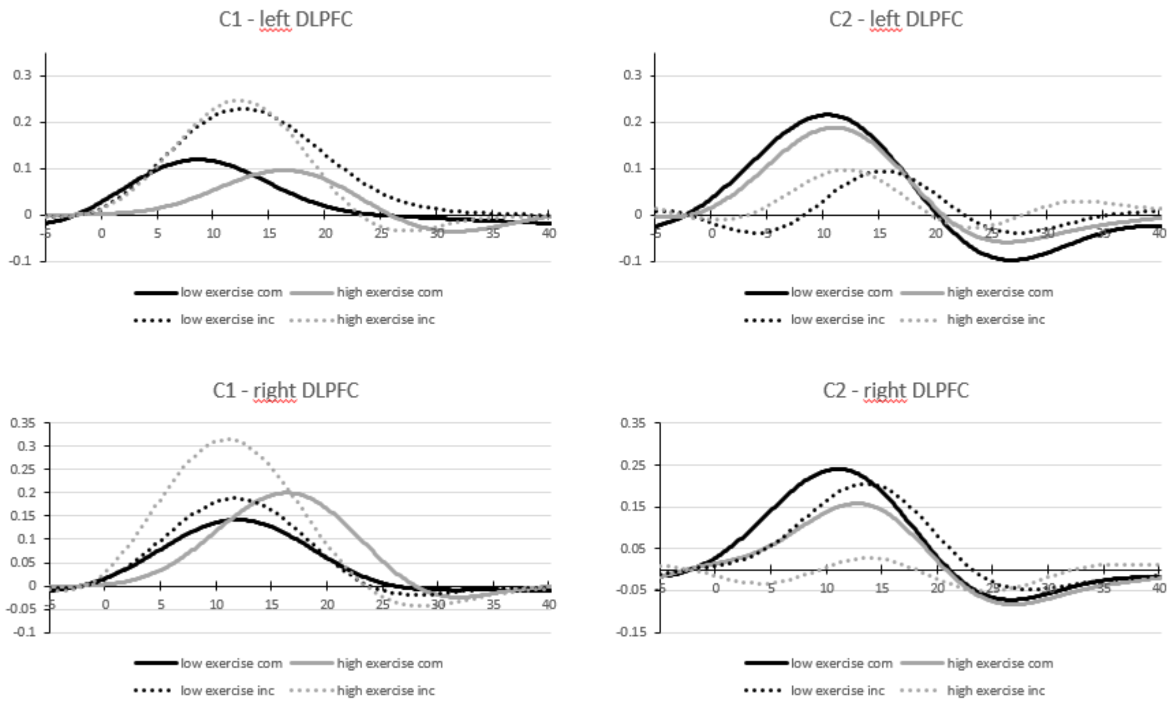

Figure S1. FNIRS waveforms of left and right dorsolateral prefrontal cortex during compatible (com) and incompatible (inc) test blocks without stress (C1) and after the stressor (C2) in participants with high and low exercise levels (in mmol/L).
